# The Group-Level Consequences of Sexual Conflict in Multigroup Populations

**DOI:** 10.1371/journal.pone.0026451

**Published:** 2011-10-24

**Authors:** Omar Tonsi Eldakar, Andrew C. Gallup

**Affiliations:** 1 Center for Insect Science, University of Arizona, Tucson, Arizona, United States of America; 2 Department of Biological Sciences, Binghamton University, Binghamton, New York, United States of America; Cajal Institute, Consejo Superior de Investigaciones Científicas, Spain

## Abstract

In typical sexual conflict scenarios, males best equipped to exploit females are favored locally over more prudent males, despite reducing female fitness. However, local advantage is not the only relevant form of selection. In multigroup populations, groups with less sexual conflict will contribute more offspring to the next generation than higher conflict groups, countering the local advantage of harmful males. Here, we varied male aggression within-and between-groups in a laboratory population of water striders and measured resulting differences in local population growth over a period of three weeks. The overall pool fitness (i.e., adults produced) of less aggressive pools exceeded that of high aggression pools by a factor of three, with the high aggression pools essentially experiencing no population growth over the course of the study. When comparing the fitness of individuals across groups, aggression appeared to be under stabilizing selection in the multigroup population. The use of contextual analysis revealed that overall stabilizing selection was a product of selection favoring aggression within groups, but selected against it at the group-level. Therefore, this report provides further evidence to show that what evolves in the total population is not merely an extension of within-group dynamics.

## Introduction

Many features of the environment influence population growth of organisms. For instance, fluctuations in local population growth are often attributed to differences in predation, disease, interspecific competition, abundance of resources and local climate [1]. Intraspecific competition can also have profound impacts. In multigroup populations, variation in these features can create population dynamics in which certain groups contribute proportionately more offspring to the next generation than others [1]. In the case of social behaviors, differences in local population growth attributed to the differential distribution of behavioral types amongst groups can influence the overall frequency of behavioral types in subsequent generations (i.e. group-level selection, see [2]).

Consider the classic example of the ‘tragedy of the commons’ (TOC) whereby individuals who exploit a shared resource are favored over their more prudent rivals within groups, despite the consequence that over-exploitation can lead to local extinction [3], [4]. In these scenarios, groups containing fewer exploitative types (or more prudent types) contribute more offspring to subsequent generations, countering the local advantage of exploitation and maintaining prudent types in the population [2]. The evolutionary consequences of differential group productivity have been well appreciated with regards to the evolution of social behaviors such as resource consumption and altruism [4], yet only recently have they been attributed to scenarios involving sexual conflict [5]–[8].

Typically in sexual conflict, the competition amongst males over females often reduces female fitness and thus local population growth [9], [10]. These population-level consequences have been demonstrated in a wide range of taxa including fruit flies *Drosophila melanogaster* [11], guppies *Poecilia reticulata* [12], [13], lizards *Lacerta vivipara* [14], grasshoppers *Stenobothrus lineatus* [15], water striders *Aquarius remigis* [6]–[8], [16], as well as in some mammals and non-human primates [17]. In these scenarios, exploitive—high harassment—males are favored over their more prudent counterparts within groups, despite these detrimental consequences to the group. Similar to the TOC, however, groups with lower sexual conflict are more productive than higher conflict groups [6], [8], [11], [16]. We have recently explored this scenario in the water strider, *A. remigis*, which is well known to engage in sexual conflict and occupy multigroup populations of moderately flowing streams. In these studies, aggressive males acquire more matings than less aggressive males within groups, but overall mating activity declines steeply with the proportion of aggressive males within groups [7]. Therefore when considering sufficient variation in aggression amongst groups, selection at the group-level can influence the landscape of selection in the overall multigroup population. One such source of this group variation in natural populations is by the conditional movement of individuals in response to local social conditions [18]–[20]. This has been previously demonstrated in freely mixing populations of *A. remigis*, whereby females dispersed away from aggressive regions to aggregate around lower aggression males, increasing heterogeneity amongst groups and strengthening group-level selection opposing aggressive mating. Aggressive males also dispersed between groups, yet did not entirely counteract female movement, resulting in the balance between within and between group selection on aggressive mating [6], [8], which likely produces the variation in aggression [21], and sex-ratio [21], [22] observed amongst pools in wild populations.

Although, we have previously demonstrated how freely mixing experimental populations provide a more accurate account of how variation at the group-level is naturally imposed by individual dispersal, the consequences of group variation have been limited to observation of mate success and not other telling measures of fitness. This is due to the artifact that in a mixing population, the group as experienced by an individual is not a constant group, but the average of all group environments experienced by that individual throughout the observational period. Therefore, tracking consequences such as female fecundity, hatching success of eggs, and nymph survival becomes problematic. In this report, we artificially imposed variation in aggression amongst isolated groups to provide a greater resolution of the group-level consequences of male aggression. We assessed various parameters responsible for population growth/group fitness in addition to the number of successful matings, including female survival, eggs production, hatching success and nymph survival. We predicted that groups higher in mating aggression would suffer greater negative consequences in local population growth (group fitness) compared to lower aggression groups. As with our previous studies, however, despite these negative group-level consequences we expected aggressive males to hold a distinct mating advantage over less aggressive males within groups. As with our previous findings in which variation in groups was artificially imposed amongst isolated pools [7], as well as in mixing populations in which group variation was naturally produced by individual dispersal [6], [8], we predicted that the balance of positive within and negative between group selection forces would produce overall stabilizing selection on mating aggression in the population, further supporting the role of multilevel selection in the evolution and mediation of sexual conflict in populations.

## Materials and Methods

Our investigation of the fitness consequences of aggressive mating required a two stage experimental procedure. The first stage identified individuals that mate during the summer (referred to as summer maters), in contrast to those that delayed mating until after the summer season, see [23]), and calculated male aggressive scores of these “summer maters” to qualify for the second experiment. The second stage altered the within-and between-group variation in male aggression and measured resulting individual and group-level fitness consequences.

### Stage 1

In total, 136 (59 male, 77 female) adult water striders were collected from White Clay Creek at the Stroud Water Research Center in Avondale PA. Each water strider was individually marked and placed in a series of eight experimental pools (125 cm diameter×12 cm water depth) in a laboratory at Binghamton University maintained at 20°C. Pools were provided with a small foam raft for resting and egg laying. They were fed frozen and then thawed small crickets, *Acheta domesticus* (<1 cm) every day *ad libitum*. Each pool was observed for 15 minutes each day for four consecutive days, recording all occurrences of mating and aggressive behaviors performed by males (table 1). Lunge-ats, lunge-jump-ons and jump-ons contributed to levels of aggression (as per [7], [24]. Overall aggression scores (*S*) per individual were calculated based on a weighted formula (see Eq. 1) of aggressive interactions performed by an individual, divided by the number of observations (*Obs*), in a simplified metric originally described in [7]. Lunge-ats (*L*), lunge-jump-ons (*L_J_*), jump-ons (*J*) and mating attempts (*M*) were weighted based on their relative contributions in influencing female resting time in a multiple regression performed in [7].

**Table 1:** Ethogram of observed behaviors.

Classification	Definition
*lunge-at*	Donor strider quickly propels itself forward towards recipient strider, possibly making contact, but not ending up on top of the recipient strider's body.
*jump-on*	Donor strider jumps off the water surface to land on top of the recipients body without traversing the space between the two striders.
*mating attempt*	A male strider lunges or jumps on a female strider and attempts to hold himself on the female's back while trying to insert his penis into the female's spermatheca.
*Mating*	A mating in which a female ceases her pre-copulatory struggles.

Descriptions of observed behaviors.



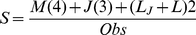
(1)


Aggression scores were normally distributed qualifying for use in parametric statistic. In addition, all successful matings, including those occurring outside of pool observations were recorded as pools were checked repeatedly for matings during the hours in which water striders are most active (12pm-3pm). Males and females that successfully mated as well as males that performed mating attempts during behavioral observations were considered active maters. In all, 30 male and 30 female water striders were identified as active summer maters and used for second stage of the experiment.

### Stage 2

After the conclusion of stage one, water striders were separated by sex for a period of 10 days so that females could lay eggs fertilized from matings occurring during the first stage of the experiment. After this period we created a total of six pools, which contained five males and five females from the previous stage. Identified male summer maters were shuffled to vary aggression within and amongst pools, creating a total of three low aggression and three high aggression pools, with the average and range of male aggression scores for each pool in the low and high aggression conditions as follows (Low: 4.98 (3.00–8.75), 3.78 (0.00–10.38), 3.80 (0.00–8.50); High: 7.34 (0.00–26.50), 7.06 (0.00–21.00), 11.96 (6.63–22.50)). Females were shuffled amongst the six pools based on their frequency of mating from the first stage of the experiment to evenly distribute mating tendency. Specifically, each pool contained three females that mated once, a female that mated twice and a female that mated greater than three times to produce an overall average matings/female for each pool of 2.2±0.10 SE. We observed matings and behavior from all of the pools on separate four occasions, while also surveying for matings in the same manner as the first stage of the experiment.

### Measurement of fitness components

We assessed local population growth/group-level fitness effects by measuring differences amongst the six pools in number of matings, eggs laid, hatching percentage, and nymph and female survival throughout the three weeks of the second experimental stage. Foam rafts provided for egg laying were removed from each pool every seventh day for a total of three times and all eggs were counted. New rafts were replaced in each pool and the old rafts were then placed in separate small eight-liter aquariums in a temperature control room maintained at 30°C. Aquariums were observed daily, and all emerged nymphs were counted and removed until all hatching was complete (two weeks time permitted for eggs to hatch). Hatching success rate of each pool was calculated for each of the three weeks from the number of hatched nymphs/number of eggs laid. Nymph survival rates were calculated using nymphs emerged from eggs collected during the first stage of the experiment. Thirty third-instar nymphs were added to each pool at the start of stage two, with another 15 nymphs added the following week. Nymph survival for each pool was calculated as the number of nymphs surviving to the end of the second experimental stage/nymphs added (45 total). Similarly, female survival for each pool was calculated as the number of females surviving to the end of the second experimental stage/initial number of females (five).

For the entirety of the second stage of the experiment, including the period while the sexes were separated, pools were provided a standardized diet to avoid the potential of high *ad libitum* diets buffering the negative fitness effects caused by male aggression. Water striders were fed every other day, with each pool receiving one cricket/adult strider plus six additional crickets to account for the nymphs. Diets were updated in the events of strider mortality.

### Analysis of group fitness

Group fitness was calculated as the product of eggs laid, hatching success and nymph survival. Considering the use of wild caught water striders, we are unaware of previous environmental conditions, such as foraging success and strategy [25], that might have produced individual differences in female fecundity. For example, in a recent collection of wild mating females (N = 42) that were placed in individual containers to lay eggs for one week, we observed a mean egg production of 54.8 eggs/week with a standard deviation of 25.5 [Eldakar et al. unpublished data]. Thus, to control for random effects due to these potential individual differences amongst females, eggs laid from the initial week were standardized based on mean eggs laid for week 1 (mean = 145) for all pools, with eggs produced in the following weeks used to determine relative changes in egg production. For example, if a pool experienced no decrease in egg production over the three weeks, then the pool egg total for each week is 145, with the overall egg total of 435. If egg production decreased by 50% between the first and second week of sampling, the relative egg count for week two would be 72.5, with the same logic applied in the following week as described above. With regards to pool fitness for a given week, egg production for that week (as described above) is multiplied by the corresponding hatching success and nymph survival for that week, revealing the projected production of future adults during that reproductive period. This standardization reduces the effects of random variation in female fitness amongst pools, and emphasizes differences amongst pools accumulated over time. A repeated measures ANOVA was used to compare overall differences in the general measure of pool fitness between high and low aggression pools, with the repeated measures component testing whether potential differences changed over the duration of the experiment. Statistics were not reported for the constituent components due to the nature of relatedness to our overall and primary factor of pool fitness. In addition, the standardization of eggs laid (and therefore number of nymphs hatched) effectively reduces the initial variance between groups to zero, invalidating statistics. Student's *t*-tests were used to test whether the dependent variables only measured once during the experiment (nymph and female survival) differed amongst the low and high aggression pools.

The fitness for individual males was considered as their relative proportion of group fitness. This was calculated as the product of the individual's proportion of matings within the group and the overall fitness of that group. Furthermore, since group fitness values (female fitness) were calculated based on standardized values from the first week of experimentation, any given male did not have a fitness advantage over any other male due to random variation in females amongst pools. Thus, the fitness differences amongst males within and between pools can be attributed to differences in aggression as measured in the study.

### Analysis of multilevel selection

The relationships among within and between group selection on aggressive mating were quantified in a separate analysis using contextual analysis [26]–[28], instead of the Price equation [29], [30]. Although useful, the Price methodology may falsely identify a covariance at the group-level as selection at the group-level, when in fact it may simply be a byproduct of direct selection at the individual level [28]. On the other hand, contextual analysis uses partial regressions to control for potential cross level byproducts and attribute group selection to only those effects on fitness that cannot be explained by effects at the individual level (see [26]–[28]). With regards to our study, we used individual male fitness as the dependent variable, with individual aggression and average aggression of the male's local pool as the independent variables respectively. Group selection was considered to occur if aggression at the group-level explains a portion of individual fitness beyond what is explained by individual male aggression score alone.

## Results

### Consequences of aggressive mating on group fitness

**Figure 1:**
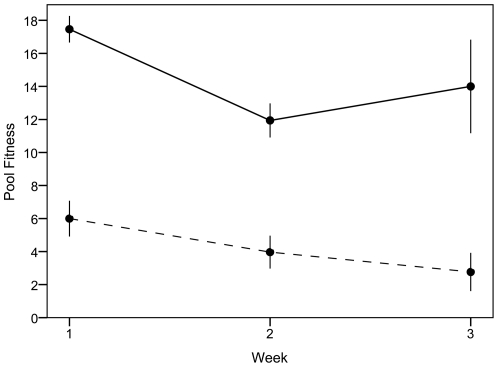
Influence of local male aggression on group fitness. Low aggression pools (solid lines) had a three times greater reproductive output than high aggression pools (dashed line), with high aggression pools experiencing no potential for population growth over the course of the experiment. Data points represent means with vertical bars indicating standard error.

Consistent with behaviors measured during stage one of the experiment, high aggression pools varied significantly from low aggression pools in aggressive behaviors (T_4_ = 2.823, *p* = 0.048), with high and low aggression pools averaging an aggression score of 43.97 (±7.92 SE) and 20.92 (±1.98 SE), respectively, per 15 minute observation period. Overall, aggressive groups were less productive than low aggressive groups (see table 2 data summaries). Low aggression pools had a significantly greater overall fitness than high aggression pools (F_1,4_ = 405.089, *p*<0.002), with low aggression pools experiencing threefold the potential population growth than high aggression pools (Fig. 1). The fitness of both high and low aggression pools decreased over the course of the three week experiment, albeit declining marginally faster in high aggression conditions (F_2,4_ = 4.745, p = 0.088). There was also a trend for nymph survival to be higher among the low aggressive pools (T_4_ = 2.405, p = 0.074), but there was no difference in terms of female survival (T_4_ = 0.866, p = 0.435).

**Table 2:** Group-level differences in fitness, and fitness components.

Pools	Low	High
*Matings*	46	30
*Total eggs laid*	1058	1069
Δ *eggs laid*	−6.0	−40.0
*Total nymphs hatched*	844	607
Δ *nymphs hatched* (%)	−11.62	−40.93
*Overall hatching success* (%)	79.77	56.78
Δ *nymph hatching success* (%)	−3.0	−0.1
*Female survival* (%)	80	60
*Nymph survival* (%)	14.81	8.14
*Total pool fitness (overall adults produced)*	102.1	32.3
Δ *pool fitness* (%)	−11.62	−40.93

Differences between low and high aggression pools. Totals represent the sum of a particular measurement over all three weeks surveyed for a treatment set. The “Δ” indicates the percentage change from week 1 to week 3 measurements for a treatment set.

### Multilevel selection-Contextual analysis

**Figure 2:**
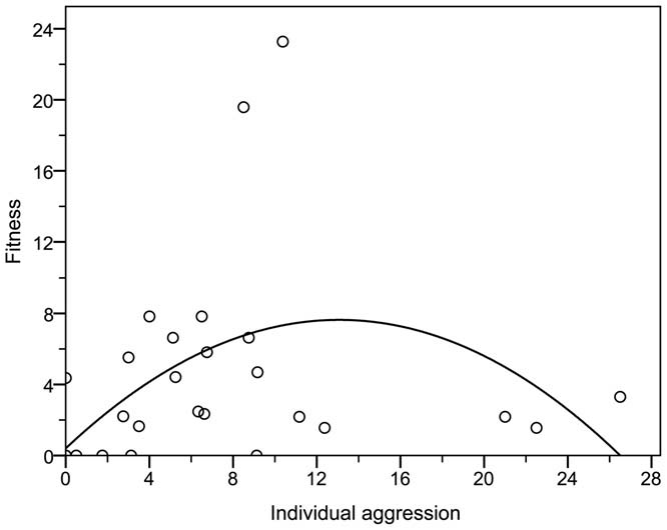
Overall stabilizing selection on male aggression in the population. Quadratic relationship between individual male aggression and fitness indicating overall stabilizing selection on male aggression in the population. Note: Five data points are overlapping, thus only 25 points are present in the figure.

**Figure 3:**
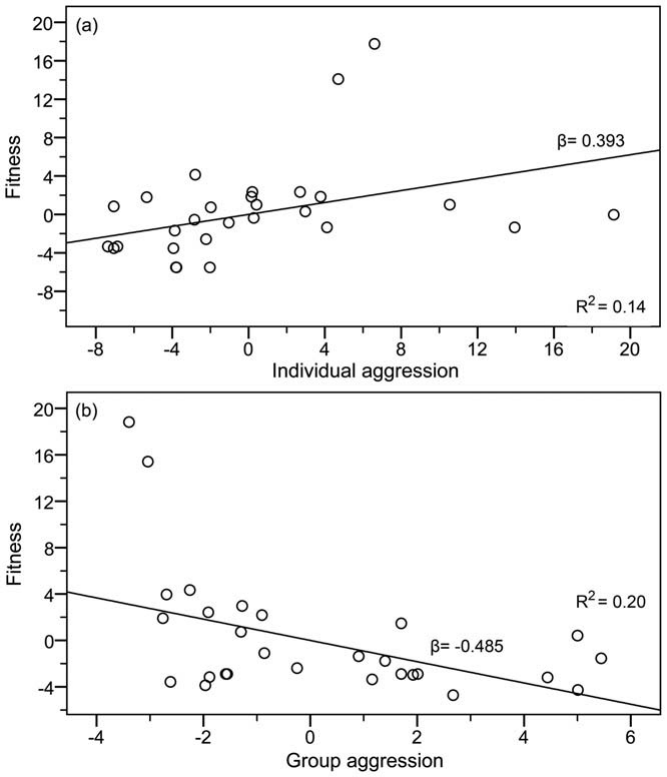
Opposing within-and between-group selection on male aggression. Plots of partial regressions (based on residuals) from contextual analysis reveals that stabilizing selection on male aggression in the population is the product of opposing within- and group-level selection. Individual male aggression score positively predicts fitness (a) while the individual's group environment of aggression predicts fitness in the opposite direction (b), when controlling for group and individual variables respectively.

As expected, aggression demonstrated a strong quadratic relationship with fitness, with a unimodal peak at a mid-range of male aggression (*R* ^2^ = 0.241, F_29_ = 4.287, *p* = 0.024), while there was no such linear trend (*R* ^2^ = 0.036, F_29_ = 1.053, *p* = 0.314), indicating overall stabilizing selection on male aggression in the multigroup population (fig. 2). Contextual analysis revealed that this stabilizing relationship was based on within-and between-group selection acting in opposite directions. Male fitness in the population was positively predicted by individual male aggression score (fig. 3a, β = 0.393, *p* = 0.044), but was negatively predicted by the group environment (average male aggression of the entire pool) (fig. 3b, β = −0.485, *p* = 0.015). These results indicate that overall stabilizing selection was the net product of selection favoring aggressive mating within groups and selection against aggression at the scale of groups, supporting our previous findings using successful matings alone [6], [8].

When running contextual analysis separately for each consecutive week, the difference in the standardized regression coefficients (β) indicate that the within group selection component of fitness declined over time (week 1 β = 0.447, week 2 β = 0.374, week 3 β = 0.336) relative to the group selection component (week 1 β = −0.507, week 2 β = −0.433, week 3 β = −0.484), indicating that group selection increased in relevance over time.

## Discussion

Previous studies on sexual conflict have shown that the competition amongst males over females can reduce female fitness, and therefore group productivity [9], [10]. However, the majority of studies on sexual conflict focus on the specific interactions between males and females, and although the group-level consequences have been somewhat appreciated [4], [5], [14]–[16], [31], few have addressed how these consequences can counter the local advantage of harmful males. In our previous reports, we have explicitly demonstrated that high aggression groups exhibit reduced mating activity compared to lower aggression groups ([6]–[8], see also [16]), countering the local within-group advantage of aggressive males. Furthermore, we have shown that in freely mixing multigroup populations, individual dispersal increases phenotypic heterogeneity amongst groups, thereby increasing the relevance of group-level consequences in mediating selection on sexual conflict [6], [8]. In the current report, we incorporated a rigid population structure, varying aggression amongst isolating groups within the range of what has been previously demonstrated in both freely mixing field [21] and laboratory populations [8], providing greater focus and additional insights to the more telling components of fitness influenced by male aggression.

Overall, less aggressive pools experienced three times the reproductive output of their high aggression counterparts, with high aggression pools experiencing virtually no population growth over the course of the experiment even without considering possible differences in adult mortality. Although the specific components of fitness could not be analyzed due to low sample size, standardization, and lack of independence between components, it is likely that even slight differences in earlier stages of development create cascading effects over time, exacerbating the consequences of aggression evident in overall group fitness. Based on previous studies demonstrating reduced female activity and foraging in the presence of aggressive males [8], [16], we hypothesize that reduced egg production is a result of poor nutrition on the part of the female, which may be indicative of the decline in egg production as females were exposed to aggressive males over greater periods of time. Furthermore, any reduced hatching rates observed in eggs from high aggression pools further contributes to this effect, combining with an already reduced egg production. This is likely to produce drastic long-term reproductive consequences on the population growth of local groups. Although we did not investigate the specific features responsible for reduced hatching success of eggs, such as fertilization rates or egg quality, future research should address which underlying physiological mechanism(s) in females and/or eggs are compromised in high aggression conditions.

Aggressive males also negatively impacted population growth at later stages in development. Nymphs developing in the high aggression pools experienced reduced survivorship compared with low aggression pools. Although low aggression pools also experienced low nymph survivor rate, cannibalism is not uncommon and has been well documented in water striders [32]–[34]. Overall high cannibalism rates across pools may also be accentuated in laboratory conditions. Compared with heterogeneous stream environments, which supply vital refugia from predation by adult water striders, laboratory systems are more homogenous and this is likely to increase the adult/nymph encounter rate. Another possible explanation for reduced nymph survivorship is the competition between nymphs and adults over food. Although we controlled for diet amongst pools, adults in high aggression pools may expend more energy and subsequently deprive nymphs of greater amounts of food in comparison to low aggression pools.

Our findings support previous research demonstrating the population-level consequences resulting from sexual conflict (see [5], [11], [14], [15], [31], [35]). This study takes the next step, however, by addressing the evolutionary implications of sexual conflict in multigroup populations. Much in the same way that altruism in social evolution is selectively disadvantageous compared to selfishness within groups, prudent (low aggression) males are also locally disadvantageous, making the explanation of their evolution problematic by only comparing fitness differentials locally within groups. Consistent with this scenario, our results suggest that despite the group-level consequences, male mating aggression is still locally favored. Frequency dependent selection still occurs within groups—with the fitness of all males plummeting with increasing aggression—but does not lead to a stable coexistence. Therefore, when selection at the local scale within groups does not correspond to selection in the overall population, something more is required to explain why aggressive males are limited in the overall population.

By continuing to incorporate a multilevel selection framework in research on sexual conflict, we demonstrated further implications of differential group productivity on the evolution of aggressive mating. The standard multilevel approach of contextual analysis revealed that the stable coexistence observed in the overall population—illustrated in the quadratic relationship between aggression and fitness—is the product of opposing directional selection within and between groups (Fig. 3). Moreover, when comparing these results weekly over the course of the experiment, group selection appears to increase in strength relative to within group selection over time. In other words, aggressive males may obtain the largest slice of the fitness pie within groups, but the shrinking of the pie reduces the size of the slice when compared to the individuals of other groups in the overall population. What evolves in the population is the phenotype that obtains the largest slice on average in the entire population, not just locally.

While the current experiment involves isolated groups, and therefore our results are immediately reflective of conditions with rigid population structures, these findings are not exclusively limited to populations of restricted migration amongst groups. As previously discussed, dispersal between groups does not necessarily ameliorate group-level consequences. When dispersal is random, heterogeneity amongst groups is typically assumed to decline, therefore reducing the strength and relevance of group selection in the population [36]. However, classic empirical studies on group selection in the flour beetle (*Tribolium castaneum*) have shown that even high levels of random dispersal (25% per generation) between local populations does not prevent genetic differentiation nor interfere with selection at the group level [37], [38]. Furthermore, the random dispersal of individuals is more the exception than the rule, as the movement of animals is often in response to environmental conditions such as food, resources, or composition of the social group [18]–[20]. When the movement of individuals is non-random, then dispersal between groups can increase group heterogeneity and therefore the strength of group selection. This is especially salient to sexual conflict as the distribution of females amongst groups can be greatly influenced by the distribution of highly aggressive males [7], [8], [15], [16], [39], [40], and is further relevant in water striders whereby both sex ratio [21], [22] and aggression [21] vary considerably amongst pools in natural populations. This has been recently supported in our aforementioned studies producing the same quadratic relationship with regards to mating frequency. In these studies, the movement of females away from local aggression created more favorable mating environments for less-aggressive males despite residing within the same interconnected multigroup population [6], [8]. Therefore, measuring differences amongst isolated groups increased our resolution of additional group-level differences in fitness (such as features which are otherwise exceedingly difficult to track, yet relevant to freely mixing populations), making a rigid population structure a necessary limitation of this study.

Although our experiment focuses on the role of the population structure, other processes may also contribute to the mediation of conflict between individuals such as density dependence as well as delayed mating. With density dependence, as the density of individuals within groups (or local populations) is reduced, investment in costly traits may exceed the benefit [41]. In the case of sexual conflict, when local densities are high the benefits of investing in costly traits to outcompete local rivals are also high. As sexual conflict reduces local population densities, however, the investment in competitive traits may prove costly as local competition is reduced, reversing local selection favoring harmful males.

In summary, by considering these group-level consequences through specific fitness related measures and their impact on the overall fitness of individuals, we were able to demonstrate that despite the local advantage of exploitative male mating strategies, these males may be limited in frequency in the total population due to their negative impact on their group (themselves included) when compared across groups. Thus what evolves in the total population is not necessarily what is favored within groups.
